# Visual-motor embodiment of language: a few implications for the neuropsychological evaluation (in Alzheimer's disease)

**DOI:** 10.3389/fnagi.2015.00184

**Published:** 2015-09-30

**Authors:** Éric Laurent, Nicolas Noiret

**Affiliations:** ^1^Laboratoire de Psychologie, Université de Franche-Comté, Université Bourgogne Franche-ComtéBesançon, France; ^2^Maison des Sciences de l'Homme et de l'Environnement, Centre National de la Recherche Scientifique, Université de Franche-Comté, Université de technologie Belfort-Montbéliard, Université Bourgogne Franche-ComtéBesançon, France

**Keywords:** vision disorders, embodied cognition, modularity of mind, enaction, enactivism, neuropsychological assessment, neuropsychological testing, sensory deficits

“*The productive combination of adjectives, nouns, verbs, and other linguistic elements corresponds to the productive combination of perceptual symbols for properties, entities, processes, and other conceptual elements*” (Barsalou, 1999, p. 594)

## Introduction

For several decades, researchers in cognitive neuroscience and cognitive psychology have developed works concerning the close relationships between “lower-level” perceptual/motor and “higher-level” conceptual/linguistic processes (Harnad, 1987; Goldstone, 1994; Barsalou, 1999; Pulvermüller and Fadiga, 2010). Some of them suggested to “reunit” perception and conception (Goldstone and Barsalou, 1998), studied the interactions between language and action (Glenberg and Kaschak, 2002; see Pulvermüller and Fadiga, 2010, for a global picture in neuroscience), or were interested in the relationships between language and other bodily (emotional) states (Glenberg et al., 2005).

On the neurophysiological and ophthalmological grounds, contemporary studies suggest that not only does Alzheimer's Disease (AD) lead to alteration in brain cortical structure and cognitive impairment, but it also conducts to deep changes in both visual system organization and vision-based performances (Tzekov and Mullan, 2013).

We recommend that both the perceptual impairment found in AD and the interactions between lower-level and higher-level cognition be taken into account by neurospychologists in order to avoid misattribution of performance deficits.

We first mention a recent research concerning language evaluation in AD and discuss main limitations of modular evaluation in that type of context. Then, we present main features of the visual “function” impairment in AD, the impacts of perceptual changes over higher-level cognition, and finally, we provide general recommendations for neuropsychological testing of higher-level cognitive “functions”.

### Linguistic evaluation in AD

Drummond et al. (2015) reported an interesting research in which language production processes were evaluated in patients with AD, amnestic mild cognitive impairment (a-MCI), and controls. In contrast with many neuropsychological tests aiming at evaluating language on the basis of simple concept production (e.g., naming), the authors developed a “narrative test,” in which participants were supposed to narrate a story from a sequence of visually presented actions. Overall, the authors found that patients with a-MCI already presented narrative deficits in comparison with the control group. Interestingly, a-MCI discursive deficits were lower than those presented by patients with AD, which may be interpreted as an intermediate level of deficiency between healthy elderly and patients with AD.

The research is interesting and allows us to examine usual practices in neuropsychology and neuropsychological research. Although the participants in this kind of research generally undergo both neuropsychological and visual (i.e., acuity) assessments, the real involvement of language “function” in the deficits found in patients with AD or a-MCI can be questioned. As we will see later, AD can lead to several visual processing impairments that influence higher-level cognitive performance so that checking for normal or corrected-to-normal visual acuity is not sufficient to control for lower-level influence on cognitive performance. Typically, whether in neuropsychology or in speech therapy, language abilities in AD are often evaluated by tests involving the visual “function”. For instance, in the naming tests, patients have to orally produce the word represented by a drawing picture. Similarly, oral comprehension tests ask patients to indicate, among several pictures, which corresponds to a word or a sentence read by the examiner. In other words, patients have to visually recognize a picture (as in the naming tests) based on an oral description. The matching category tests also require patients to choose—among several visual items—the one that is semantically associated with a target item. Finally, tests that focus on graphic abilities (e.g., dictation, free writing, writing description) also rely on visual “functions”.

When performances are altered in the tests such as those described above, any earlier level of information processing can be involved (Greene, 2005). Although they are mainly employed to evaluate language, these tests can also reflect visual “function” impairment. The semantic recognition of drawings or pictures implies that patients rely on good visual acuity, color vision, contrast sensitivity, and oculomotor processing. Misinterpretations of AD patient troubles may arise if visual performances are not taken into account (and controlled for in statistical analyses).

### The visual “function” in AD

If cognitive—and especially memory—disorders are a hallmark of AD, it is less widely known but well established, that many visual processes are also altered at multiple levels of the nervous system in AD.

The lens (equatorial supranuclear cataracts), the retina (loss of ganglion cells, narrowing of venous blood column), macula (volume decrease), the retinal nerve fiber layer (reduction in thickness at the optic nerve head), the optic nerve (widespread axonal degeneration of the retinal ganglion M-cells), the lateral geniculate nucleus (demyelination, amyloid plaques especially in parvocelullar layers), the superior colliculus [amyloid plaque and neurofibrillary tangle (NFT) accumulation], the pulvinar (amyloid plaques and neuritic plaques), the visual cortex—possibly at later stages of the disease—(neuronal loss, amyloid plaques, and NFT, especially in early-onset forms) have been found to be affected in AD (see Tzekov and Mullan, 2013, for an excellent synthesis).

Psychophysical measures have also revealed noteworthy differences between patients with AD and controls. Visual acuity might be decreased in AD, as a function of disease severity, and/or under low luminance conditions. Color perception—at least in the blue-violet spectrum—is altered in AD, and visual field measures—when possible with AD patients—have revealed field constriction with deficits being more severe in the inferior part of the visual field (potentially because of the distribution of senile plaques and NFT in the visual cortex; Tzekov and Mullan, 2013). Contrast sensitivity (CS) is also reduced in both patients with AD and patients with MCI. Clearest evidence of differences in CS as a function of group (AD, MCI, Cognitive complaints without performance deficits, Controls) has been found in the upper right visual field using frequency-doubling technology, and CS has been regarded as a potential biomarker of AD (Risacher et al., 2013). Finally, both depth (Mendez et al., 1996) and motion (Gilmore et al., 1994; see Fernandez et al., 2013, for an electrophysiological approach) perception abilities are impaired in AD (see also Mandal et al., 2012, for a global picture).

Several studies demonstrated oculomotor processing impairment in AD, even at early stages of the disease or in mild cognitive impairment (Peltsch et al., 2014; Pereira et al., 2014; Molitor et al., 2015). Increased reaction time to trigger saccades, difficulty to inhibit saccadic reflex, and decreased smooth pursuit velocity, acceleration, and accuracy, have been consistently reported (Boxer et al., 2006, 2012; Garbutt et al., 2008; Crawford et al., 2013). Eye movements involved in visual exploration/search are also impaired in AD (Rösler et al., 2000, 2005; Mosimann et al., 2004; Molitor et al., 2015). For instance, when they were supposed to search for a number among 79 letters randomly distributed on a screen, patients with AD had deficits in target detection and detection time, associated with more fixations and longer fixation duration (Rösler et al., 2000). Mosimann et al. (2004) found that during a clock-reading task, patients with AD displayed fewer fixations at the end of each clock hand, a significant delay before their first fixation landed inside these regions of interest, longer fixations, and smaller saccade amplitudes.

Not only do the reviewed studies lend support for an impairment of basic visual anatomy, physiology, and behaviors, but they also suggest that higher-level cognition can be influenced by visual impairment in AD.

### Interactions between visual and higher-level cognitive processes

As rightly noted by Tzekov and Mullan (2013), psychophysical evaluations in AD should be carried out with caution because of the potential influence of cognitive (and affective) variables—such as compliance with the instructions, assignment comprehension, and memorization, vigilance required during testing—over the measured performances. In the other way round, the numerous changes that affect vision and eye movements in AD should be taken into account while evaluating later and higher-level processes. This should also be the case of language production tasks. If visual acuity (which is the simplest but most controversial psychophysical measure to identify low-level visual impairments in AD, see Tzekov and Mullan, 2013, p. 419) is generally controlled for, other visual or visuomotor variables are scarcely taken into account when testing language performances.

This state of affair, which tends to undermine the granted weight of lower-level perceptual-motor processes in cognitive neuropsychological testing, is all the more problematic that previous empirical studies reported clear interferences, not only between visual impairment and Benton's Facial Recognition, or Visual Form Discrimination tests (Kempen et al., 1994), but also between visual impairment and higher-level-cognition evaluation. For instance, Killen et al. (2013) found that visually impaired elderly individuals scored lower than controls in both the vision-dependent items of the Mini-Mental-State-Examination (MMSE) and the Clock drawing test (CDT), but not when vision-independent items of the MMSE and the CDT were proposed. Even if the visual impairments under consideration were rather severe (e.g., macular degeneration, glaucoma), the data suggest that attention should be paid to the control of visual impairment when higher-level cognition is tested. Note that the reduction in macular volume has been reported in AD and has been found to be related to cognitive performance (as indexed by the MMSE; Iseri et al., 2006).

A recent review of the literature outlined the overlap between cataract and cognitive impairment (Jefferis et al., 2011). Wood et al. (2010) reported impaired performance in older adults across three cognitive tests (the digit symbol substitution test, trail making test A and B, the Stroop color word test) when cataract conditions were simulated.

As another example, by manipulating the stimulus strength of each item of several tests through contrast sensitivity function filtering (i.e., low-degraded, medium-normal, and high-enhanced stimulus-strength conditions), Cronin-Golomb et al. (2007) demonstrated that the modification of stimulus strength altered performances in several tests in AD. They found that performances in *letter identification, word reading, picture naming*, and *face discrimination* decreased more in AD patients in comparison with healthy elderly in the low-degraded condition. Interestingly, AD patients improved their performances to a level equal to their healthy counterparts when stimulus strength was enhanced.

In this context, and given the extent of visual alteration in AD, much care should be taken when considering language performances, especially if the task requires conceptual production from visual stimulation.

## Conclusion: the need for more systematic evaluations of visual processes and more systemic reasoning in neuropsychology

Taken as a whole, data concerning the effects of AD over visual processes and those demonstrating the influence of visual impairment on higher-level cognition suggest that controlling for visual impairments in patients with AD could provide critical information to attribute capacity loss to appropriate processing levels. Neuropsychologists know well the strong time constraints that often feature clinical neuropsychological testing. However, when and where possible, some measures should be performed or taken into account (when they are provided by an ophthalmologist) in order to control for any effect of lower-level process (e.g., visual or visuomotor-encoding processes) impairment over higher-level cognitive “functions” (e.g., language production). This is critical when the protocol involves *the perception and the interpretation of a visual scenario*. Disentangling the language production impairments from other disorders in this kind of settings implies to (i) more thoroughly examine potential differences in visual and eye movement performances between patients and controls through the use of appropriate visual/visuomotor tests, and (ii) consider those performances as covariates in any further group comparison concerning cognitive abilities, namely when the cognitive test requires the processing of visual information. This is needed to better understand and characterize the cascade of alterations associated with AD; especially because AD patients “*are less likely than healthy elderly individuals to report vision problems to their physicians*” and that “*sensory deficits can be hidden and may masquerade as higher order deficits*” (Gilmore et al., 2004).

Beyond the specific case of language, the proposed approach asks basic questions about our conception of cognitive processes. The discussed effects remind us how much cognition is situated, grounded, embodied in specific perceptual (Goldstone and Barsalou, 1998; Barsalou, 1999) and perceptual-motor (Laurent, 2014) systems, which allow recursive processes, conceptual elaboration, and the enaction of what is more classically regarded as modular “cognitive functions.”

### Conflict of interest statement

The authors declare that the research was conducted in the absence of any commercial or financial relationships that could be construed as a potential conflict of interest.
